# Transcriptomic analysis highlights epigenetic and transcriptional regulation during zygotic embryo development of *Pinus pinaster*

**DOI:** 10.1186/1471-2229-13-123

**Published:** 2013-08-30

**Authors:** José J de Vega-Bartol, Marta Simões, W Walter Lorenz, Andreia S Rodrigues, Rob Alba, Jeffrey F D Dean, Célia M Miguel

**Affiliations:** 1 iBET - Instituto de Biologia Experimental e Tecnológica, Apartado 12, 2780-901 Oeiras, Portugal; 2 Instituto de Tecnologia Química e Biológica, Universidade Nova de Lisboa, Av. da República, 2780-157 Oeiras, Portugal; 3 Warnell School of Forestry and Natural Resources, The University of Georgia, Athens, GA 30602, USA; 4 Monsanto Company, Mailstop CC4, 700 Chesterfield Parkway West, Chesterfield, MO 63017, USA

**Keywords:** Conifer embryogenesis, Epigenetics, Gymnosperm, Transcriptomics, Transcription factor

## Abstract

**Background:**

It is during embryogenesis that the plant body plan is established and the meristems responsible for all post-embryonic growth are specified. The molecular mechanisms governing conifer embryogenesis are still largely unknown. Their elucidation may contribute valuable information to clarify if the distinct features of embryo development in angiosperms and gymnosperms result from differential gene regulation. To address this issue, we have performed the first transcriptomic analysis of zygotic embryo development in a conifer species (*Pinus pinaster*) focusing our study in particular on regulatory genes playing important roles during plant embryo development, namely epigenetic regulators and transcription factors.

**Results:**

Microarray analysis of *P. pinaster* zygotic embryogenesis was performed at five periods of embryo development from early developing to mature embryos. Our results show that most changes in transcript levels occurred in the first and the last embryo stage-to-stage transitions, namely early to pre-cotyledonary embryo and cotyledonary to mature embryo. An analysis of functional categories for genes that were differentially expressed through embryogenesis highlighted several epigenetic regulation mechanisms. While putative orthologs of transcripts associated with mechanisms that target transposable elements and repetitive sequences were strongly expressed in early embryogenesis, PRC2-mediated repression of genes seemed more relevant during late embryogenesis. On the other hand, functions related to sRNA pathways appeared differentially regulated across all stages of embryo development with a prevalence of miRNA functions in mid to late embryogenesis. Identification of putative transcription factor genes differentially regulated between consecutive embryo stages was strongly suggestive of the relevance of auxin responses and regulation of auxin carriers during early embryogenesis. Such responses could be involved in establishing embryo patterning. Later in development, transcripts with homology to genes acting on modulation of auxin flow and determination of adaxial-abaxial polarity were up-regulated, as were putative orthologs of genes required for meristem formation and function as well as establishment of organ boundaries. Comparative analysis with *A. thaliana* embryogenesis also highlighted genes involved in auxin-mediated responses, as well as epigenetic regulation, indicating highly correlated transcript profiles between the two species.

**Conclusions:**

This is the first report of a time-course transcriptomic analysis of zygotic embryogenesis in a conifer. Taken together our results show that epigenetic regulation and transcriptional control related to auxin transport and response are critical during early to mid stages of pine embryogenesis and that important events during embryogenesis seem to be coordinated by putative orthologs of major developmental regulators in angiosperms.

## Background

Embryogenesis is a crucial period in the life cycle of most plant species. Molecular aspects of reproductive biology and embryo development have been widely studied in model angiosperms, which diverged from the gymnosperms more than 300 million years ago to follow a distinct evolutionary pathway within the Spermatophyta [1]. As such, striking differences are visible during reproduction and embryogenesis, of which the double fertilization in angiosperms *versus* single fertilization in gymnosperms is a major example. Important differences observed during conifer embryo development also include: (1) nuclear duplication without cytokinesis during proembryogeny instead of the initial asymmetric cell division commonly observed in the zygote of angiosperms; (2) the frequent occurrence of polyembryony; (3) the differentiation of tube cells during early embryo development; and (4) the formation of multiple cotyledons during late embryo development [2]. These differences imply that differences in the molecular regulation of embryo development must exist between the two groups of plants. *Pinus* represents the largest genus within the coniferous family Pinaceae, and among the gymnosperms is also the most widespread genus of trees in the northern hemisphere. A substantial amount of information on the repertoire of transcribed genes in several pine species is available in a variety of databases [3,4]. However, most of the transcriptomics studies using this information to date have focused on stress resistance/tolerance and wood development [5-8]. The most comprehensive study of transcript profiling in *Pinus* embryos has been conducted in *P. taeda*, where approximately 68,700 ESTs have been generated from somatic and zygotic embryos [9]. The authors suggested that differences between the embryo developmental pathways in angiosperms and gymnosperms are primarily the result of differential control of spatial and temporal gene expression along with the expression of unique proteins. However, the expression dynamics of genes transcribed at different stages of embryo development was not studied. In *A. thaliana*, Spencer et al. [10] concluded that in terms of overall transcriptional profiles of several embryo stages, temporal expression differences were more significant than spatial differences. Very recently, differential gene expression during somatic embryogenesis of Norway spruce (*P. Abies*) has been probed using a microarray of *P. taeda* sequences, which allowed identification of molecular events regulating putative processes associated with pattern formation and differentiation [11]. Most studies in conifers up to now have relied on the use of somatic embryos but, while somatic embryogenesis is a useful experimental model system for studying embryology in conifers, it is also recognized that the conditions provided during *in vitro* culture, such as the provision of synthetic auxins, can have an impact on transcript profiles [12]. Most developmental responses to auxin appear to be mediated through changes in gene expression and external application of auxin cause profound effects in plant growth and development [13]. Moreover, abnormal morphology has been reported for somatic embryos of *P. pinaster*, which are routinely induced on auxin-containing medium [14]. Although zygotic embryogenesis is the model against which somatic embryogenesis is typically compared, zygotic embryo development has rarely been studied because the isolation of zygotic embryos from immature conifer seeds is technically challenging, especially at early stages of embryo development [15].

In the present study, we have characterized the transcriptome of *P. pinaster* zygotic embryos isolated at different developmental stages, from early embryogenesis to embryo maturation, using a loblolly pine cDNA microarray containing approximately 25,000 unique cDNAs [7], with the aim of identifying transcripts and biological processes associated with specific developmental stages, and emphasizing early embryo development. To our knowledge, this is the first genome-wide study of transcript profiles across zygotic embryogenesis in pines. Our approach uncovered major regulatory genes with putative roles in epigenetic and transcriptional control of key developmental processes. Comparative transcriptomic analyses against an *A. thaliana* embryogenesis model [16] further highlighted these regulatory functions.

## Results

### Microarray analysis of the *Pinus pinaster* developing embryo transcriptome

**Figure 1:**
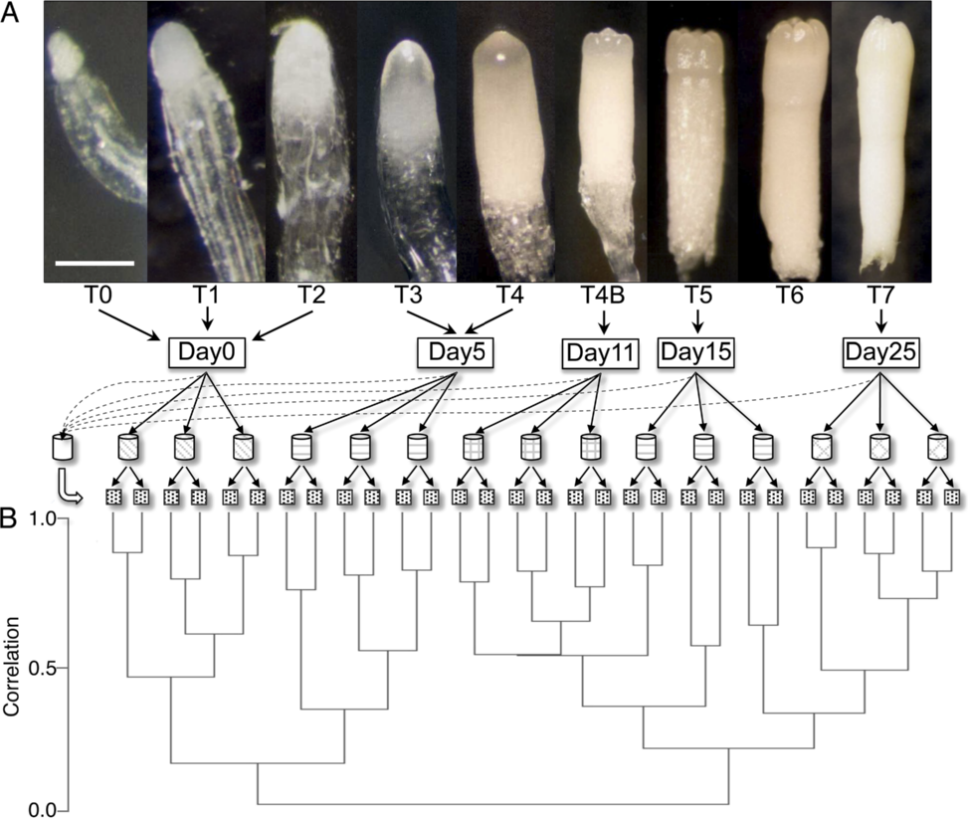
**Microarray hybridization. (A)** Staging system (T0 to T7) used for *Pinus pinaster* zygotic embryo development [15], showing how samples were divided into five developmental groups/time-points representing early embryogenesis (T0 to T2), pre- cotyledonary (T3 and T4), early cotyledonary (T4B), late embryogenesis (T5) and mature embryo (T7). Bar: T0 and T1 = 300 μm; T2, T3 and T4 = 400 μm; T4B = 800 μm; T5, T6 and T5 = 0.1 cm. Three biological replicates of each sample harvested on Day 0 to Day 25 and two technical replicates were used for hybridization with the reference sample, which consisted of a pool containing equal amounts of total RNA from all five time points. **(B)** Cluster analysis of the thirty replicates generated using MeV with Pearson correlation and average linkage.

Transcript level dynamics during *P. pinaster* zygotic embryogenesis were analyzed using the PtGen2 cDNA microarray (GPL11184) for hybridization of samples representing five sequential periods of embryo development. Based on previous studies in which maritime pine embryo development was monitored [15,17], we isolated dominant zygotic embryos at five time points representing consecutive stages of embryo development grouped as early, pre-cotyledonary, early cotyledonary, cotyledonary and mature embryos (Figure 1). The PtGen2 microarray contains 25 848 (26 496 total features minus buffer blanks and duplicate spots) amplimers of cDNA clones derived from thirty-six cDNA libraries constructed exclusively from loblolly pine (*P. taeda*) root and needle tissue; no embryonic tissue was utilized in its construction [7,18]. The use of this array for cross-species hybridization with target samples from diverse *Pinus* species, including *P. pinaster,* has been previously demonstrated [7,18]. In fact, loblolly pine cDNA arrays have been successfully used also for gene expression analysis in other conifer genera [11,19]. In total, 30 microarray slides were used in our study (Figure 1A). Box plots of the expression values pre- and post-normalization confirmed that the data were successfully normalized (Additional file 1). The quality of the microarray datasets was demonstrated by verifying reproducibility among replicates by hierarchical cluster analysis using Pearson correlation and average linkage (Figure 1B). Samples harvested at the same time point clustered together and separated from samples of other time periods, with the exception of Day15. Technical replicates always clustered together and their correlation values ranged from 0.7 to 0.85. Overall, a close relationship between technical replicates and among samples harvested at the same time point (biological replicates) was observed, whereas samples from distant time points showed greater variability.

In order to improve subsequent annotation, *P. taeda* ESTs corresponding to the 3′ and 5′ ends of cDNAs spotted on the PtGen2 array were used in a BLASTX search against the SustainPineDB, which contains non-duplicated set of transcripts for *P. pinaster* (Unigenes). A total of 10 922 spots were aligned to the same unigene using either the 3′ or the 5′ end sequences. Another 6911 spots for which only a single end sequence was available (the 3′end in most of cases) aligned with a single unigene. There were 3105 spots that aligned to different unigenes depending on whether the 3′ or 5′ end sequence was used, and these were consequently associated with 6210 unigenes. In all cases, duplicated spots aligned to the same unigene, and in a few cases different clones aligned to the same unigene. In total, 20 938 spots were aligned with 14 996 different unigenes from SustainPineDB (Additional file 2). By contrast, no significant alignment was found in SustainPineDB for 5294 of the microarray spots. In such cases, the *P. taeda* 3′ EST sequence, when available, was used during annotation. Alternatively, the 5′ EST sequence was used. The 14 996 recovered unigenes plus the 5294 *P. taeda* EST end sequences were annotated by comparison against the NCBI protein database (nr) using BLASTX (E-value < 1e-10). Orthologs were found for 13 280 unigenes and 3482 cDNA clones (Additional file 3). Identity (matches/alignment length) distribution peak was at 75%. Of the top hits, 47.4% corresponded to *Picea sitchensis,* 10.3% to *Vitis vinifera* and 3.4% to *Ricinus communis.* The remaining hits did not correspond to more than 2.5% for any one species (Additional file 4). Gene Ontology terms (http://www.geneontology.org/) [20] were subsequently associated with 12 659 unigenes and EST sequences using Blast2GO (Additional file 3). The annotated GO terms ranked from level 2 to 11, and were concentrated around level 7. Most of the sequences that could not be annotated were shorter than 1 kb (Additional file 4). We also used BLASTX (E-value < 1e-10) to look at *A. thaliana* orthologous proteins corresponding to each of the *P. pinaster* unigenes. A total of 13 265 unigenes aligned with 7732 *A. thaliana* proteins, thus providing Plant Ontology, pathway and gene family annotations based on TAIR mappings (http://www.arabidopsis.org) (Additional file 3).

### Time-course analysis of gene functional categories during embryo development

**Figure 2:**
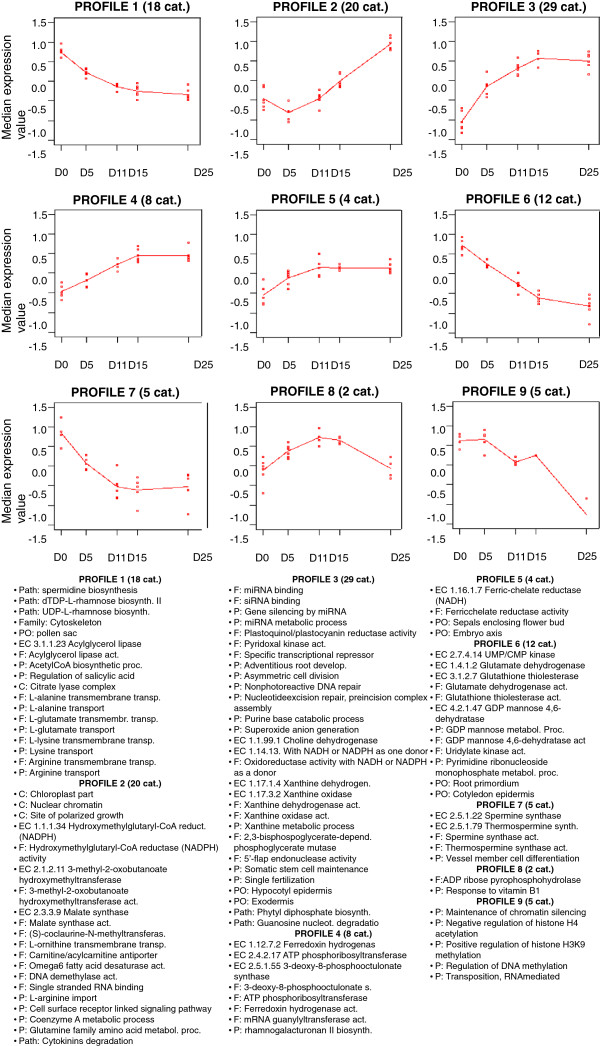
**Clustering of functional categories that showed similar expression profiles during *P. pinaster *zygotic embryogenesis.** Functional categories include gene ontologies, plant ontologies, enzyme codes, pathways, families and structures annotations. The expression of a category is represented by the median expression values of the genes annotated within that category. Dots indicate the distribution (mean and quartiles) of the median values for the categories included in each profile/cluster. Functional categories are listed in the inset.

To get an overview of the processes and functions significantly associated with different stages of zygotic embryo development we first performed a functional assessment of expressed transcripts using the regression model in maSigFun for the analysis of time-course microarray experiments. In this analysis, functional categories whose genes significantly changed transcript levels with the same pattern over time indicate a high level of co-expression within them and a relationship to the embryogenic process [21]. Differential expression (FDR < 0.01) was noted in 103 functional categories that clustered into nine profiles (Figure 2). Some profiles showed similar trends in expression at the same developmental stages. For example, profiles 1, 6 and 7 were up-regulated during early embryogenesis, while profiles 3, 4 and 5 were down-regulated during the same period. However, the profiles all showed different fold-changes that distinguished them from each other at these stages. The genes assigned to each category based on their ontological annotations are described in Additional file 3. The number of categories within each profile ranged from 2 to 29. In addition to GO biological processes, functions and cell components, the identified functional categories included 18 EC numbers, six pathways, one gene family (cytoskeleton in profile 1), and seven plant ontology terms. However, it should be noted that EC numbers were redundant to GO terms in all cases.

Amino acid transport and metabolism, as well as nucleotide metabolism, were among the most prevalent functional categories up-regulated during early embryogenesis, but showed a gradual decrease at subsequent stages of development (profiles 1 and 6). Enzymes at important branch points between carbon and nitrogen metabolism, such as glutamate dehydrogenase, or involved in the biosynthesis of central metabolites for carbon and energy metabolism, such as acetyl coenzyme A, or in glutathione metabolism, such as glutathione thiolesterase, were also prevalent in these transcript profiles. Functions related to GDP- mannose metabolism, particularly GDP-mannose 4,6-dehydratase activity, which is associated with GDP-L-fucose biosynthesis, were also up-regulated during early embryogenesis. Cytoskeleton gene family members were up-regulated in early embryogenesis, as were pathways for the biosynthesis of cell wall components, such as dTDP/UDP-L-rhamnose (Figure 2).

Profile 9, in which functional categories are highly expressed during early to mid- embryogenesis, but drastically down-regulated towards the mature embryo (Day25) was of particular interest. All co-expressed categories identified in this profile appeared to be associated with mechanisms of epigenetic regulation, including maintenance of chromatin silencing, regulation of histone acetylation or methylation, and regulation of DNA methylation. In contrast, processes mediated by small RNAs appeared prevalent as a subset of co-expressed categories in profile 3, with an expression trend that increased towards late embryogenesis. Included in this profile were miRNA metabolic process, siRNA and miRNA binding, and gene silencing by miRNA. A high number of co-expressed categories followed a similar trend, including functions related to purine catabolic recycling activities involving xanthine dehydrogenase and xanthine oxidase enzymes, and the guanosine nucleotide degradation pathway. DNA replication and repair processes, which are indicative of a high DNA replication rate, were also identified in this profile together with biosynthesis of structural components, such as phytyl diphosphate. In profile 2, which differs from profile 3 by its sustained increase in expression through the mature embryo stage, processes related to fatty acid metabolism were clearly over-represented, but other mechanisms, such as those related to polarized growth, chromatin organization and fine regulation of cytokinin, were also present.

### Transcriptional profile analysis during embryo development

For the analysis of individual genes, differentially transcribed sequences were extracted using maSigPro [22]. The method first adjusts a global regression model to identify sequences that are differentially transcribed with respect to time, after which a variable selection strategy is applied to study differences between groups and find significantly different profiles. A total of 3081 spots were differentially transcribed during embryo development in *P. pinaster* (FDR < 0.001; Additional file 5). Of these, 384 spots were associated to two unigenes, 2210 spots were associated to one unigene, and 487 spots could not be associated to any unigene in SustainpineDB and the EST was used instead. However, some spots mapped to the same unigene and the number of unique unigenes was 2633, therefore the total number of differentially transcribed sequences considered in our analyses was 3120 (2633 unigenes and 487 ESTs). Orthologs were found in the NCBI nr database for 2814 of these sequences, while 2161 had orthologs in the *A. thaliana* TAIR10 database. We were able to associate GO terms to 2485 of these sequences (Additional file 5).

**Figure 3:**
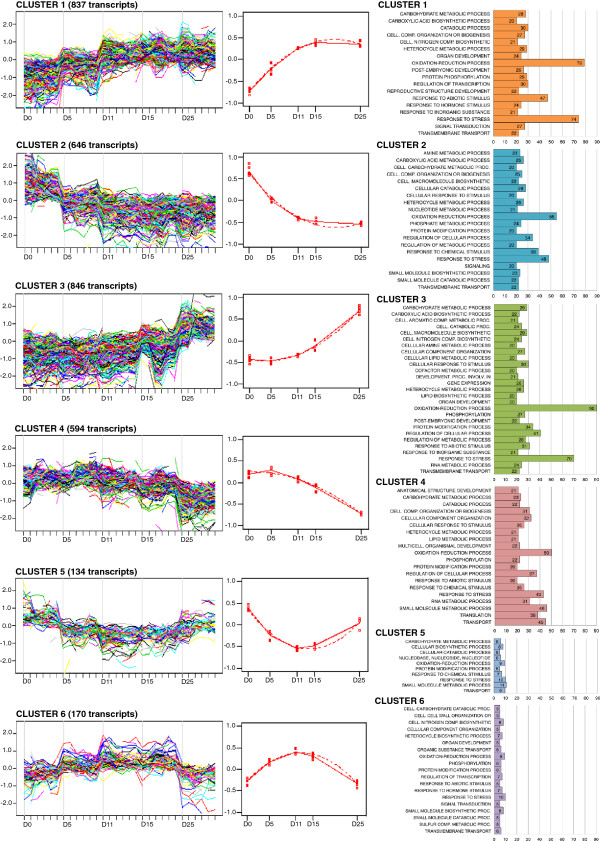
**Clustering of differentially expressed genes according to their expression profiles during *P. pinaster *zygotic embryogenesis, and representative gene ontology terms in each cluster.** Transcripts having similar expression profiles, which were differentially expressed in time according to maSigPro analysis, were clustered together, and a representative median expression profile was inferred from the expression of all the genes in each cluster. For each cluster, the number of transcripts in each gene ontology term is indicated in a bar graph.

Based on their transcript levels across the five developmental time points, the 3081 spots could be grouped in 6 clusters, formed by 796, 631, 812, 555, 125 and 162 spots, respectively. The spots mapped to 837, 646, 846, 594, 134 and 170 unigenes and ESTs, respectively, to sum up 3227 differentially transcribed sequences (Figure 3). Some of the unigenes that mapped to multiple spots fell into more than one cluster. The six transcript profiles could be further grouped by pattern into up (clusters 1 and 3), down (clusters 2 and 4), up-down-up (cluster 5), and down-up-down (cluster 6) clusters describing changes from early embryogenesis to the mature embryo. We found that 1683 (52.2%) transcripts were up-regulated while 1240 (38.4%) were down-regulated across the embryo development time course. The remaining 170 (5.3%) and 134 (4.2%) transcripts were either up- or down-regulated, respectively, during the intermediate time points (clusters 5 and 6), which correspond to the pre-and early-cotyledonary stages of development.

Changes in transcript profiles during developmental transitions that included the most extreme time points, Day0 → Day5 and to Day15 → Day25, were more evident and involved a larger number of genes than changes at the intermediate time points, especially Day11 → Day15. This is not particularly surprising considering the shorter time-window between Day11 and Day15, as well as the close developmental proximity between early-cotyledonary and cotyledonary embryos, which differ mainly regarding the enlargement of pre-formed organs.

We evaluated the *biological process* GO term distribution in each cluster (Figure 3). Terms were joined in a superior level when the number of transcripts was smaller than 20. The analysis of GO terms showed that the metabolic process *oxidation-reduction* is over- represented in most clusters, followed by *response to stress*. The exceptions were clusters 5 and 6, which included relatively few annotated genes. However, certain GO terms were identified only in specific clusters, suggesting their association with defined periods of embryo development. For example, *anatomical structure development* (which included *organ* and *shoot morphogenesis*, and *photomorphogenesis*) and *multicellular organismal development* (includes embryo development) were both associated with early embryogenesis (cluster 4). *Post-embryonic development*, *developmental process involved in reproduction,* and *reproductive structure development* (which included seed, fruit, embryo and flower development) were constrained to clusters 1 and 3, which represented transcripts accumulating mostly from Day11-15 up to the mature embryo. O*rgan development* transcripts (which included *post-embryonic organ*, shoot and root development, and leaf senescence) were also present in clusters 1 and 3, but also cluster 6. Although the more generic processes *cellular response to stimulus*, *response to chemical*, and *stress* or *abiotic stimulus* were present in all clusters, *response to hormone stimulus* was associated only with mid- and late embryogenesis stages (clusters 1 and 6), while *response to inorganic substance* was exclusive to late embryogenesis (cluster 1). Eighty-three (83) differentially expressed transcripts were only found in gymnosperms (annotated in Additional file 5). These corresponded with 53 unigenes and 26 ESTs, as several transcripts were associated with more than one unigene. On average, about 3% of the sequences in each cluster corresponded to gymnosperm-specific sequences, and they were equally distributed between early and late embryogenic stages. Few of these sequences showed similarity to NCBI accessions.

### Differential transcript profiles associated to epigenetic regulation

We focused our analysis of differentially transcribed genes on the identification of putative master regulators that might drive expression of the embryo transcriptome, with an emphasis on epigenetic regulators and transcription factors that could potentially impact development. A list of 24 selected genes whose annotations suggest involvement in epigenetic regulation is presented in Table 1. Several associated with chromatin remodelling, including histone post-translational modifications, DNA methylation, and regulation of small RNA biogenesis and processing, were up-regulated at different stages of embryo development. However, 71% of the transcripts in Table 1 were found in clusters 1 and 4, which display opposite patterns of transcription during early and late embryo development.

**Table 1:** Differentially transcribed genes implicated in epigenetic regulation

**Cluster**	**Gene ID**	***Pp *unigene**	***At *Locus**	**E-value**	**Annotation**
1	6.2.9.22	732	AT1G08460	2E-141	Histone deacetylase 8 (HDA8)
1	3.1.21.21	1009	AT3G44680	0	Histone deacetylase 9 (HDA9)
1	9.3.15.18	25311	AT1G09700	2E-43	dsRNA-binding protein 1 (DRB1), HYPONASTIC LEAVES 1 (HYL1)
1	2.2.5.12	38	AT1G48410	0	ARGONAUTE 1 (AGO1)
1	9.4.14.10	9118	AT1G48410	0	ARGONAUTE 1 (AGO1)
1	1.4.19.11	5048	AT5G21150	3E-109	ARGONAUTE 9 (AGO9)
1	7.2.18.7	3877	AT2G23380	2E-151	CURLY LEAF (CLF), INCURVATA 1 (ICU1), SDG1, SET1
1	1.2.3.11	401	AT5G22750	2E-155	DNA/RNA helicase protein RAD5
1	2.2.17.8	62665	AT3G20550	6E-84	SMAD/FHA domain-containing protein DAWDLE, DDL
1	9.3.17.23	15368	AT5G14170	1E-164	SWIB/MDM2 domain superfamily protein CHC1
1	4.1.9.18	60482	AT5G14170	1E-164	SWIB/MDM2 domain superfamily protein CHC1
2	10.2.21.21	28419	AT1G01040	0	ABNORMAL SUSPENSOR 1 (ASU1), CARPEL FACTORY (CAF), DCL1,
					DICER-LIKE1 (DCL1), EMBRYO DEFECTIVE 60 / 76 (EMB60 / 76),
					SHORT INTEGUMENTS 1 (SIN1), SUSPENSOR 1 (SUS1)
3	10.2.13.22	1158	AT1G77300	5E-27	H3-K4 specific histone methyltransferases, ASH1 HOMOLOG 2 (ASHH2)
3	4.4.11.7	441, 7804	AT4G38040	7E-150	Exostosin family protein
3	8.4.10.21	7514	AT3G17590	3E-75	Transcription regulatory protein SNF5 homologue, BUSHY GROWTH (BSH)
4	5.3.8.1	17585	AT5G03740	4E-19	Histone deacetylase 2C (HD2C)
4	7.1.7.5	18705	AT1G77540	5E-30	H3/H4 histone acyl-CoA N-acyltransferase
4	1.4.19.18	25013	AT2G47210	4E-124	myb-like transcription factor family protein
4	1.3.3.10	2926	AT5G66750	0	CHROMATIN REMODELLING 1 (CHR1), DECREASED DNA
					METHYLATION 1 (DDM1), SOMNIFEROUS 1 (SOM1)
4	8.2.9.24	4432	AT4G16280	9E-73	RNA-mediated chromating silencing protein, FLOWERING TIME CONTROL
					PROTEIN FCA
4	6.4.2.10	5264	AT3G57300	1E-157	INO80 ortholog
5	8.1.4.5	739, 26140	AT1G57820	0	Zinc C3HC4-type RING finger protein, ORTH2, VARIANT IN
					METHYLATION 1 (VIM1)
6	11.2.17.19	19820	AT5G26040	5E-136	Histone deacetylase 2 (HDA2)
6	2.3.12.22	3694	AT5G04940	5E-123	SU(VAR)3-9 homolog 1 (SUVH1)

Four putative histone deacetylase (*HDA*) genes were identified among the differentially expressed transcripts. While two of them, *HDA8* and *HDA9*, showed increasing transcription from early embryogenesis up to the cotyledonary embryo stage (cluster 1), *HD2C*, which belongs to a class found only in plants [23], showed an opposite transcript profile (cluster 4). The fourth, *HDA2*, was up-regulated in mid-embryogenesis especially at the early cotyledonary stage (cluster 6).

Genes related to the methylation of histones were also identified with different transcript profiles. A putative ortholog of the *A. thaliana* polycomb-group (Pc-G) gene *CURLY LEAF* (*CLF*), part of the Polycomb Repressive Complex 2 (PRC2), was up-regulated in mid- to late embryogenesis (cluster 1). A putative ortholog of *ASH1 HOMOLOG 2* gene (*ASHH2*), which encodes a protein with histone lysine N-methyltransferase activity implicated in the regulation of gene expression via H3K36 trimethylation [24], showed increasing transcription towards maturation (cluster 3). Finally, a putative *SU(VAR)3-9 homolog 1* (*SUVH1*) was specifically up-regulated in early cotyledonary embryos (cluster 6). Several putative orthologs to SWI2/SNF2 chromatin-remodelling ATPases that modulate the accessibility of genomic regions to the transcriptional machinery [25] were differentially expressed. Two of them were found in cluster 1, namely *CHC1* and *RAD5*, while a third one, *BUSHY GROWTH* (*BSH*), which is a putative ortholog of yeast SNF5 (subunit of the SWI/SNF chromatin remodelling complex), was up-regulated in late embryogenesis (cluster 3). Cluster 4 contained a differentially expressed ortholog of *INO80* which is a member of the SNF2 superfamily of ATPases.

Transcripts for genes related to DNA methylation, *DECREASE IN DNA METHYLATION 1* (*DDM1*) and *DNA methyltransferase 1-associated protein* (*DMAP1*), were up-regulated during early embryogenesis through the pre-cotyledonary embryo stage (cluster 4). A putative ortholog of *VARIANT IN METHYLATION 1* (*ORTH2/VIM1*) was found in cluster 5.

Finally, several transcripts with homology to known regulators of small RNA biogenesis, processing, and function, were also differentially regulated during embryo development. Transcripts encoding a putative DAWDLE (DDL) were identified in cluster 1. Putative orthologs of *HYPONASTIC LEAVES1* (*HYL1*) and *DICER-LIKE1* (*DCL1*) were found in cluster 1 and 2, respectively. Additionally, two putative *ARGONAUTE* transcripts, *AGO1* and *AGO9*, were found in cluster 1. With an opposite transcript profile, we also identified a *FLOWERING LOCUS CA* (*FCA*) putative ortholog in cluster 4.

### Transcription factors involved in consecutive embryo stage-to-stage transitions

**Figure 4:**
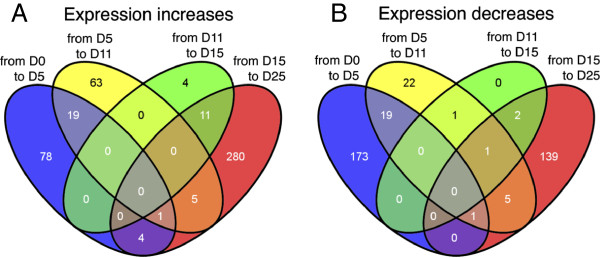
**Venn diagrams of genes regulated between two consecutive stages.** The number of genes showing a fold-change >2 between consecutive stages is shown. For each transition, genes showing increasing **(A)** or decreasing **(B)** expression between consecutive stages are represented in different diagrams.

Transcripts displaying a fold-difference ≥ 2 between consecutive embryo developmental stages were examined to identify genes that might be relevant for a specific period of development (Additional file 6). Consistent with the time-course analyses of transcript profiles and functional categories, the most dramatic changes in expression were noticed in the transition from Day0 → Day5, where 173 transcripts were specifically down-regulated and 78 transcripts were up-regulated, and in the transition from Day15 → Day25, where 280 and 139 transcripts were specifically up and down-regulated, respectively (Figure 4). Only four transcripts were specifically differentially regulated (up-regulated) in Day11 → Day15 period (Figure 4), and no genes were up-regulated simultaneously in Day0 → Day5, Day5 → Day11 and Day11 → Day15 transitions, suggesting major differences between the transcriptomes of early and late stage embryos.

Focusing on genes involved in transcriptional regulation, we identified transcripts annotated as likely transcription factors (TFs) in Table 2. Among the 23 TFs identified, about 2/3 were up-regulated in a specific developmental transition, with Day15 → Day25 showing the highest number of differentially expressed TFs. The bHLH, followed by the NAC and MYB transcription factor families, were most represented in our analyses. The best characterized putative TF found to be up-regulated in early embryogenesis (Day0 → Day5) was KANADI 2 (KAN2), a member of the GARP transcription factor family. Up-regulation during early and mid embryogenesis was also observed for a putative ortholog of *AINTEGUMENTA* (*ANT*) (Table 2), while a putative *YABBY2* transcript seemed to be important in the transition from early cotyledonary to cotyledonary stage. Putative orthologs for three *NAC* transcripts, namely *NAM*/*NARS2*, *ARABIDOPSIS NAC DOMAIN CONTAINING PROTEIN 75* (*ANAC075*) and *ATAF1*, were up-regulated in the Day15 → D25 transition (Table 2), while a putative *bHLH* transcript, *LEUCINE RESISTANT 3* (*ILR3*), was strongly up-regulated in Day15 → Day25. Worth noting was the strong down-regulation from Day0 → Day5 (20.9 fold) of a putative *AUXIN RESPONSE FACTOR*, *ARF16*, which may serve to underscore the relevance of auxin response mechanisms in the early stage embryos. In this early stage, a putative ortholog coding for a bZIP TF, *FLORAL TRANSITION AT THE MERISTEM 3* (*FTM3*)*,* and a putative *WRKY28* were found to be down-regulated. TFs that were specifically down-regulated in the Day15 → Day25 transition included members of the bHLH superfamily, such as a putative *FAMA* homologue. Additionally, a homologue of the PLATZ family of plant-specific TFs, and *HAP5B*, whose roles are poorly or not yet characterized were also down-regulated at this period.

**Table 2:** Transcription factor genes showing a transcript level fold-change ≥ ; 2 between consecutive stages of embryo development

**Cl**	**Gene ID**	***Pp *unigene**	***At *Locus**	**E-value**	**Annotation**	**Up-regulated**	**FC**	**Down regulated**	**FC**
1	6.3.22.5	20408	AT4G38620	7E-72	R2R3 MYB protein 4 (MYB4)	D0 → D5	2.7	-	-
						D5 → D11	2.4		
1	12.1.13.17	7865	AT3G54390	2E-45	Sequence-specific DNA binding protein	D0 → D5	2.2	-	-
1	6.4.7.23	1862	AT1G68920	8E-56	Basic helix-loop-helix (bHLH) DNA-binding superfamily protein	D0 → D5	2.1	-	-
6	8.2.2.4	1030	AT1G32240	8E-41	KANADI family transcription factor 2 (KAN2)	D0 → D5	2.1	-	-
1	6.1.20.1	27680	AT4G37750	2E-96	AINTEGUMENTA (ANT)	D5 → D11	2.4	-	-
3	10.3.2.2	2388	AT1G08465	1E-41	YABBY family protein 2 (YAB2)	D11 → D15	2.2	-	-
3	3.3.10.21	57615	AT1G12540	3E-13	Basic helix-loop-helix (bHLH) DNA-binding superfamily protein	D11 → D15	2.0	-	-
						D15 → D25	4.2		
3	1.1.20.3	37794	AT3G49950	5E-46	GRAS family transcription factor	D15 → D25	2.0	-	-
3	2.3.14.1	25514	AT1G52880	4E-53	ARABIDOPSIS NAC DOMAIN CONTAINING PROTEIN 18 (ANAC018), NO APICAL MERISTEM/SEED MORPHOLOGY 2 (NAM/NARS2)	D15 → D25	2.3	-	-
3	2.4.19.23	17360	AT1G22640	1E-150	MYB-type transcription factor 3 (MYB3)	D15 → D25	2.3	-	-
3	2.4.7.21	2808	AT4G29230	7E-98	NAC-domain protein 75 (ANAC075)	D15 → D25	2.4	-	-
3	3.1.19.21	12873	AT5G47390	4E-71	MYB-like transcription factor	D15 → D25	2.0	-	-
3	3.2.1.2	16071	AT1G01720	7E-75	NAC-domain protein 2 (ANAC002), ATAF1	D15 → D25	2.7	-	-
3	4.4.11.10	270	AT3G20910	6E-31	Nuclear factor Y subunit A9 (NF-YA9)	D15 → D25	2.0	-	-
3	5.2.18.9	16530	AT5G54680	1E-54	Basic helix-loop-helix (bHLH) 105 (BHLH105), LEUCINE RESISTANT 3 (ILR3)	D15 → D25	4.1	-	-
2	1.3.17.22	8657	AT2G18160	2E-14	Basic leucine-zipper 2 (BZIP2), FLORAL TRANSITION AT THE MERISTEM 3 (FTM3)	-	-	D0 → D5	3.3
2	5.1.1.2	37241	AT4G18170	2E-27	WRKY transcription factor 28 (WRKY28)	-	-	D0 → D5	2.1
5	5.3.10.21	2451	AT4G30080	3E-151	Auxin response factor 16 (ARF16)	-	-	D0 → D5	20.9
6	1.4.9.14	22607	AT3G24140	1E-51	bHLH superfamily protein FAMA	-	-	D15 → D25	2.2
4	10.3.12.3	17529	AT2G12646	3E-67	PLATZ transcription factor family protein	-	-	D15 → D25	3.0
6	3.3.17.13	7237	AT1G65620	1E-56	ASYMMETRIC LEAVES 2 (AS2)	-	-	D15 → D25	3.2
4	4.3.3.12	4970	AT1G56170	4E-56	Nuclear factor Y subunit C2 (NF-YC2), HAP5B	-	-	D15 → D25	2.7
4	4.4.11.2	18019	AT3G19500	2E-31	bHLH DNA-binding superfamily protein	-	-	D15 → D25	2.5
1	6.4.7.23	1862	AT1G68920	8E-56	bHLH DNA-binding superfamily protein	-	-	D15 → D25	2.1

### Comparative time-course analysis of differentially expressed transcripts during embryogenesis in *P. pinaster versus A. thaliana*

We compared the transcript profiles for genes involved in embryo development in *A. thaliana* and *P. pinaster* in order to search for correlations between the levels of putative orthologous transcripts. For each developmental stage that we considered as equivalent between species, genes were plotted in a scatter graph using the *A. thaliana* and *P. pinaster* expression values as coordinates (Additional file 7). Linear regression analysis demonstrated that the highest correlations were in the first (Day0 and globular embryos) and last (Day25 and mature embryos) stages, while the lowest correlations were in the third stage (Day11 and torpedo embryos).

To compare transcript profiles between the two species, the fold-changes in transcript levels at each stage *versus* the average value throughout embryogenesis in each species was quantified for each data series. The transcript profiles for 224 genes in *P. pinaster* and *A. thaliana* had a Pearson correlation higher than 0.90 (Additional file 8). An enrichment analysis using Agrigo [26] of the 206 genes that had GO annotations in the *A. thaliana* TAIR10 database revealed that seven biological functions were significantly over- represented (FDR < 0.001; Additional file 9), specifically *catabolic process*, *cellular component organization*, *developmental process*, *multicellular organismal process* and several *metabolic process* categories.

Among the genes with highly correlated transcript profiles between both species we found: (i) six *EMBRYO DEFECTIVE* genes, namely *GNOM* / *EMB30* (AT1G13980), *S- ADENOSYL-L-HOMOCYSTEIN HYDROLASE 1* / *EMB1395* (AT4G13940), *SECY HOMOLOG 2* / *EMB2289* (AT2G31530), EMB2296 (AT2G18020), RIBOSOMAL PROTEIN 1 / EMB2207 (AT2G18020) and *CYTOKINESIS DEFECTIVE 1 / EMB101* (AT2G39770); (ii) several differentially expressed genes within the *developmental process* category that have been previously shown to affect embryo development in *A. thaliana* via transcription and regulatory processes, such as *SKP1-Cullin*/*CDC53-F-box* (also *LEAF CURLING RESPONSIVENSS*, *LCR*) and *ETHYLENE-INSENSITIVE 2* (*EIN2*; AT5G03280); and (iii) two relevant epigenetic regulators, *MULTICOPY SUPPRESSOR OF IRA 1* (*MSI1*; AT5G58230) and *UBIQUITIN CARRIER PROTEIN 2* (*UBC2*; AT2G02760).

### Validation of microarray data

**Figure 5:**
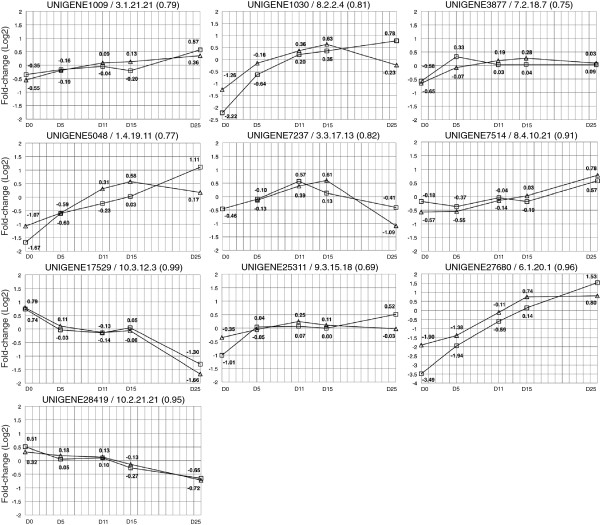
**Validation of microarray transcript profiles.** Fold-changes for selected transcripts obtained by microarray analysis and RT-qPCR are shown for each developmental time point. Insets represent the profiles based on microarray M-values intensity ratios (triangles) or RT-qPCR relative expression normalized values (squares).

To validate the microarray expression data, a set of ten differentially transcribed genes putatively involved in epigenetic and transcriptional regulation (Tables 1 and 2) were analysed using RT-qPCR. Selected genes showed different transcription profiles during zygotic embryo development. Five genes belonged to cluster 1, two genes to cluster 6, and one gene to each of the clusters 2, 3 and 4 (Figure 3). Four of the genes (Unigenes 1030, 7237, 27680 and 17529) were transcription factors. Microarray green/red intensity ratios (M values) and RT-qPCR transcription levels were obtained for each gene (Additional file 10). The magnitudes of both data series were equalized or normalized calculating the fold-change between each time point and the average value, then log_2_ transformed as shown in Figure 5. Correlation between microarray and RT-qPCR data was demonstrated by high Pearson correlation coefficients that ranged from 0.69 (Unigene 25311) to 0.99 (Unigene 17529).

## Discussion

Zygotic embryo isolation is a major challenge in most plant species, especially in the early stages of embryo development [16]. Our method allowed for rapid separation of embryos by stage and verification of structural integrity before freezing, which enabled collection of sufficient amounts of tissue for RNA isolation. In this way, we were able to perform the first genome-wide transcript profiling study that covers a wide time-window of zygotic embryo development in a conifer species.

Many genes required for embryo development are not embryo-specific as their basal functions are needed throughout the plant’s life cycle [27]. Le et al. [28] reported that out of 16,000 genes active throughout *A. thaliana* seed development, only 289 are seed-specific. However, significant quantitative changes in gene activity occur across specific developmental stages. In fact, each stage of seed development has a characteristic gene set that is either specific or up-regulated with respect to genes active at other stages [28]. We found that oxidation-reduction metabolic processes were over-represented in most clusters, which is indicative of high metabolic activity taking place in the developing embryos. Maintenance of cellular redox homeostasis by redox metabolites, such as glutathione, likely plays an important role in the context of embryo development. In fact, glutathione metabolism was highlighted in our analyses of differentially expressed functional categories (*glutathione thiolesterase activity*) and by the high number of transcripts putatively encoding glutathione transferases. These observations would appear in line with previous reports that the abundance of glutathione in proliferating cells is critical for shoot and root meristem development through roles in auxin transport and signalling [29]. Moreover, it has been shown that manipulation of glutathione metabolism during *in vitro* embryogenesis can affect embryo yield and quality, i.e. an environment with elevated levels of reduced glutathione results in increased numbers of immature embryos during somatic embryogenesis induction, while a more oxidized environment promotes embryo development [30].

Conifers are a major group within the gymnosperms, and are interesting subjects for studying embryogenesis due to distinguishing characteristics that when compared to angiosperms may reveal unique genes and gene networks that could further illuminate plant embryo development and its evolutionary implications. In our analyses, we found that approximately 3% of the differentially regulated transcripts appeared unique to gymnosperms with most putatively coding for unknown or uncharacterized proteins. While these unknown proteins might play important roles in conifer embryo development, still it appears that embryogenesis is mainly accomplished by the coordinated activities of a similar set of transcripts in both angiosperms and gymnosperms, as has been previously suggested [9]. The features that differentiate embryo development in these two plant groups probably result primarily from differential gene regulation. Thus, we have focused our study on the analysis of regulatory mechanisms of gene expression that play important roles during plant development, namely epigenetic control and transcriptional control by transcription factors.

### Epigenetic regulation pathways across embryo development

Covalent modification of histones, DNA methylation, chromatin-remodelling enzymes and small RNAs, among other factors, play a central role in gene expression by modulating access to DNA and defining distinct chromatin states that ultimately determine selective readout of the genomic sequence [31].

Maintenance of chromatin silencing, specific histone post-translational modifications, and regulation of DNA methylation and transposition, appeared as co-regulated functions during early embryogenesis in our time-course analysis of functional categories across pine embryogenesis. In early embryogenesis, co**-**regulated functional categories as well as the identified differentially regulated transcripts, pointed to the importance of gene silencing mechanisms related to the control of transposable elements (TE). In fact, DNA methylation and heterochromatin maintenance were highlighted by both the analysis of co**-**regulated functional categories and the up**-**regulation of a putative *DDM1*, which is a key regulator of heterochromatic formation in *A. thaliana* required for TE**-**specific DNA methylation [32]. An identical transcript profile was observed for a putative FCA, recently implicated in the regulation of RNA sequences related to transposons, retrotransposons, and dispersed repeats that are normally silenced by the RNA**-**directed DNA methylation pathway [33]. Also, a putative *ORTH2*/*VIM1*, a mediator of DNA methylation status implicated in the establishment/maintenance of chromatin structure during cell division [34], was up**-**regulated during early embryogenesis in pine, although it showed a distinct transcript profile across embryo development being down**-**regulated specifically at mid embryogenesis.

*DCL1*[35], a homologue of which was up**-**regulated in Day0 samples, has been recently suggested to play a role in TAS**-**derived small interfering RNA**-**triggered DNA methylation by directly processing *TAS* gene transcripts [36]. DCL1 is required for cell differentiation events as early as the eight**-**cell stage *A. thaliana* embryos [37], where it participates in early embryonic patterning. Through its action on miRNA biogenesis, DCL1 prevents the accumulation of miRNA targets that promote differentiation during later stages of embryogenesis, namely transcription factors [37].

Evidence for negative regulation of histone H4 acetylation together with positive regulation of H3K9 methylation found in the time**-**course analysis of functional categories suggests a trend towards transcription repression during early embryogenesis. Specifically, H3K9 trimethylation, but not H3K9 methylation or dimethylation, is found in highly expressed genes [38]. In contrast, hyperacetylated histones are generally associated with gene activation, whereas hypoacetylated histones are related to gene repression [23]. Our results suggest that different groups of genes are being targeted for epigenetic regulation during embryogenesis through the action of different classes of deacetylases, e.g. a putative HD2C in early embryogenesis *versus* putative HDA8 and HDA9 in late embryogenesis. Inhibition of histone deacetylases (HDACs) in *A. thaliana* is known to affect embryo development as well as the expression of seed**-**associated genes, including transcription factors [39,40]. In another conifer, Norway spruce, Uddenberg et al. [41] reported a similar behaviour, suggesting a connection between changes in acetylation patterns and the levels of embryogenesis**-**related gene expression.

A homologue of *SUVH1*, which encodes a H3 lysine**-**9 specific histone**-**lysine N**-** methyltransferase, was up**-**regulated specifically in early cotyledonary embryos (cluster 6), suggesting a possible role in the organization of transcriptionally repressive chromatin [42]. Altogether these results suggest that different well**-**known mechanisms of gene silencing are active during early embryo development in pine.

From the mid to late embryogenesis stages, large chromatin remodelling events also seem to take place in conifer embryos as suggested by significant increases in transcription of several apparent chromatin**-**remodelling ATPases, such as putative CHC1, RAD5 and BSH. Another gene up**-**regulated in mid**-** to late embryogenesis (cluster 1) was a homolog of *CLF*, a member of the polycomb**-**group (Pc**-**G) proteins, which regulate many developmental processes in plants and animals by repressing gene expression in a cell**-**specific manner via trimethylation of histone H3 lysine 27 (H3K27me3) [43]. *CLF* was initially characterized as a suppressor of floral homeotic genes [44], but other genes such as *CUP****-****SHAPED COTYLEDON 2* (*CUC2*) and *PIN1* have been described as Pc**-**G target genes [45,46]. *CUC* genes, for example, which are regulated by *PIN1*, are crucial for the establishment of the embryonal shoot apical meristem and the formation of two separated cotyledons by presumably preventing cell proliferation and cotyledonary outgrowth in the intercotyledonary regions [47]. The *Picea abies PaNAC01*, an orthologue of *CUC*, is also regulated by polar auxin transport and it is associated with differentiation of the shoot apical meristem and formation of separated cotyledons [48]. Consistent with these findings, loss of H3K27me3 in *Arabidopsis* was shown to be associated with misregulation of genes involved in auxin responses [49]. It seems likely that in our results, the observed differential regulation of *CLF* and other chromatin**-**remodelling genes is related to the regulation of some of these processes.

In what concerns small RNA pathways, several transcripts with homology to known regulators of small RNA biogenesis, processing, and function were differentially expressed during pine embryo development, including a putative *DDL*, mostly up**-**regulated from mid to late embryogenesis, that could act in the biogenesis of miRNAs and endogenous siRNAs. DDL does not affect transcription of miRNAs directly but acts through other proteins, such as DCL, by facilitating their access or recognition of pri**-**miRNAs [50]. The processing of miRNAs from longer primary transcripts (pri**-**miRNAs) requires the activity of several proteins, including DCL1 and the double**-**stranded RNA**-**binding protein, HYPONASTIC LEAVES1 (HYL1) [35,51,52], for which a putative ortholog was found up**-**regulated in the same developmental periods as the putative DDL.

Argonaute (AGO) proteins are part of the RNA-induced silencing complex (RISC) that bind small RNAs and cause gene silencing. Two putative *AGO* transcripts similar to *AGO1* and *AGO9*, were highly represented in the late pine embryo transcriptome. AGO9 belongs to a phylogenetic clade in which all members recognize 24-nucleotide small interfering RNAs and act to silence TEs and other repetitive sequences at the transcriptional level [53]. In contrast, orthologs of AGO1 are primarily mediators of miRNA activities [54,55], although AGO1 is also involved in the production of RDR6-dependent siRNAs [56,57]. In summary, different gene silencing mechanisms seem to be more active in opposite stages of pine embryo development. In early embryogenesis, mechanisms that target TE and repetitive sequences appear dominant, while PRC2-mediated repression of genes involved in specific developmental processes, such as formation of cotyledons, seems to be more relevant during late embryogenesis. In contrast, genes associated with sRNA pathways were found to be differentially regulated across all stages of embryo development.

### Transcriptional regulators and auxin-mediated events

When chromatin structure allows expressed TFs to gain access to their binding sites, these proteins play a master role in the regulation of gene expression. A putative ortholog of *ARF16* showed a dramatic decrease in transcription from the early embryo to precotyledonary stage of development, suggesting it could play a major role in early pine embryogenesis. ARFs are key regulators of auxin-modulated gene expression [58] that activate or repress target genes by binding to the promoters of early auxin response genes. ARF16 and ARF10 were shown to repress *WOX5* transcription and restrict it to the *A. thaliana* root quiescent center [59] where it is required to maintain pluripotent columella stem cells [60]. Interestingly, in addition to being expressed in early embryogenesis, the putative *ARF16* transcript profile in pine (cluster 5) also showed an increase from early cotyledonary to mature embryos, which is a profile similar to the one described for *ARF16* in *A. thaliana* embryos [61]. Another gene up-regulated in early pine embryos was a putative ortholog of *AUXIN RESISTANT1* (*AUX1*), which encodes an auxin influx carrier. Together with ATP Binding Cassette subfamily B (ABCB) transporters and PIN proteins, AUX1 carriers are primary coordinators of polar auxin transport. A homolog of *N-MYC DOWNREGULATED-LIKE 1* (*NDL1*), which plays a role in modulating auxin transport, possibly by regulating auxin transport carrier proteins like PIN2 and AUX1 [62], was also up-regulated in early stage pine embryos.

If these gene products serve conserved functions in gymnosperm embryogenesis, then the interplay between auxin and transcription factors with defined spatial and temporal expression patterns is critical for the establishment of the pine embryo patterning. For example, the putative *ARF16* expression pattern in pine embryos seems to be consistent with what has been described for *ARF16* in *A. thaliana*, where it is involved in establishment of apical-basal patterning by participating in initiation of the root apical meristem formation in an early stage of embryogenesis.

KANADI protein (KAN2) plays an important part in early angiosperm embryogenesis, presumably by modulating the flow of auxin through regulating polar expression of PIN proteins [63]. Our studies suggest a KAN2 homolog may be important in the transition from early stage pine embryogenesis to the precotyledonary embryo stage. Interestingly, differential regulation of a homolog of PIN3 (Additional file 6), an auxin efflux carrier, and of a homolog of GNOM (cluster 4, Additional file 5), important for the recycling of PIN proteins between endosomal compartments and the plasma membrane [64], observed during the same period of development is in agreement with such a role for the putative KAN2. At the heart stage of *A. thaliana* embryogenesis, *KAN2* as well as *KAN1* and *KAN3,* display a similar expression pattern in the abaxial basal portion of emerging cotyledon primordial [63]. The same authors proposed that pattern formation along the central–peripheral axis results from interplay between auxin and the KANADI and Class III HD-Zip transcription factors.

Eshed et al [65] proposed that initial asymmetric leaf development, regulated primarily by mutual antagonism between KANADI and PHABULOSA (PHB)-like genes, is translated into polar YABBY expression, which subsequently contributes both to abaxial cell fate and abaxial/adaxial juxtaposition-mediated lamina expansion. A putative *YABBY* gene family member, *YABBY2*, was significantly up-regulated in pine from early cotyledonary stage onwards, which could be consistent with involvement in abaxial cell fate determination and leaf lamina growth along the abaxial–adaxial boundary [66,67]. Another transcript, a putative *AS2/LOB*, encoding a plant-specific protein involved in the determination of adaxial-abaxial polarity in leaf primordia [68-70] and repression of class 1 KNOTTED-like homeobox (KNOX) genes [71], was down-regulated in the Day15 → Day25 transition. Pine genes likely involved in the formation and function of apical meristems were also differentially expressed, including a putative *NARS2/NAM*, which was significantly up- regulated in late pine embryogenesis. *NARS2/NAM* has been implicated both in the formation of organ boundaries and embryonic shoot apical meristem. For example, the *A. thaliana CUC1*[72] and *CUC2*[73] as well as the petunia *NO APICAL MERISTEM (NAM)*[74], are expressed in boundaries between floral organ primordia and in the boundary between the cotyledons. However, mutations in *CUC1*, *CUC2*, and *NAM* also affect the initiation of the shoot apical meristem [72-75]. *AINTEGUMENTA (ANT)* has been implicated in the regulation of shoot apical meristem function [76]. A putative pine *ANT* homolog may be important for the transition from pre-cotyledonary to early cotyledonary embryos, as suggested from its up-regulation during this period. Plants containing mutations in *ANT* genes exhibit increased sensitivity to disruptions in polar auxin transport [77], suggesting that its role during embryogenesis might be related to auxins.

### Correlation between profiles of putative orthologs between *A. thaliana* and *P. pinaster* embryogenesis

By matching embryo stages between two distantly related species, comparative analyses between the model angiosperm *A. thaliana* and *P. pinaster* lead us to conclude that several important processes in embryo development are conserved between angiosperms and gymnosperms. In fact, a few genes known to be essential for *A. thaliana* embryo development, including *EMBRYO DEFECTIVE* genes, and respective putative pine orthologs were highlighted by our analyses. One of these genes, *GNOM* (*EMB30*), plays a pivotal role in early *A. thaliana* embryogenesis being required for embryo patterning [78]. Its function in endosomal recycling of PIN1 (and PIN2) to the basal plasma membrane is fundamental for apical-basal polarity establishment during embryogenesis. Through tissue-specific expression of *GNOM* in *gnom* mutant embryos, Wolters et al. [78] showed that both apical and basal embryo organization depend on *GNOM* provascular expression, and proposed that GNOM-dependent PIN relocalization (cell-autonomous) and sink-driven auxin transport (non cell-autonomous) trigger the initiation of auxin transport routes in embryonic pattern formation. Additional relevant genes involved in auxin-mediated responses were identified in this study by comparative analysis. For example, LCR has been shown to negatively regulate several auxin-responsive genes during leaf development [79], and EIN2 is involved, together with AUX1 and GNOM, in establishing a concentration gradient of auxin in the root tip important for root-hair positioning within the epidermal layer [80].

Finally, there were two relevant epigenetic regulators whose transcript levels decreased over time in highly similar fashion, suggesting equivalent roles in developing *A. thaliana* and *P. pinaster* embryos: *MSI1* and *UBC2*. MSI1 is a core protein of the plant PRC2 complex required for normal seed development [81]. MSI1 is needed to maintain the correct temporal and organ-specific expression of homeotic genes, including *AGAMOUS* and *APETALA2*, as well as to establish epigenetic marks, such as H3K4 di-methylation and H3K9 acetylation, in SOC1 chromatin [82-84]. UBC2 is involved in the regulation of ubiquitination of histone H2B and control of flowering time in *A. thaliana* through modulation of FLOWERING LOCUS C/ MADS AFFECTING FLOWERING chromatin [85].

## Conclusions

Taken together, our results indicate that characteristic transcriptional changes are associated with each developmental period, as has been previously observed for *A. thaliana* embryogenesis [10,28], and suggest that major events during embryogenesis are orchestrated by putative orthologs of regulators of these processes in angiosperms. Our analyses were limited to some extent because the stages of embryo development used in this study did not cover certain periods where major events that distinguish angiosperm and gymnosperm embryogenesis take place, such as the first zygote divisions after fertilization and polyembryony. Thus, further work should focus on the earliest stages of pine zygotic embryo development, before dominant embryos are identified. An expanded knowledge of conifer genomes will also be essential to further understand the molecular basis for characteristic features of embryogenesis in gymnosperms. Additional insight into the molecular regulation of embryo development in these species will have great utility for the improvement of conifers and their vegetative propagation through somatic embryogenesis.

## Methods

### Plant material

Immature female cones from *Pinus pinaster* Ait. were randomly collected from open- pollinated trees growing in a clonal orchard established by grafting between 1970 and 1975 at Escaroupim National Forest, Portugal (longitude 8°44’W, latitude 39°4’N). Cone harvesting was performed at five different time points from July to August 2007, selecting from 3–5 trees at each date. Immediately after cone harvest on each date seeds were removed from the cones and opened to expose the megagametophytes. These were dissected using fine forceps and a scalpel under a stereomicroscope to excise the dominant embryo. Isolated embryos were quickly evaluated for developmental stage and immediately frozen in liquid N_2_ into different pools depending on stage. The five time points were designated as Day0 for the first harvesting date and Day5, 11, 15 and 25 according to the day of harvest after Day0. Following a previously described embryo staging system [15], Day0 included embryos in developmental stages T0, T1 and T2 (early embryo stages), Day5 included stages T3 and T4 (pre-cotyledonary embryos), Day11 included stage T4B (early cotyledonary embryos), Day15 included stage T5 (cotyledonary embryos) and Day25 included stage T7 embryos (mature embryos) (Figure 1A). Several separate pools, each containing 30–60 zygotic embryos for each stage, were prepared for each time point and kept in separate tubes to serve as biological replicates in subsequent analyses.

### RNA isolation and amplification

Total RNA was extracted from each pool of frozen zygotic embryos using the RNeasyPlant Mini Kit (Qiagen, Valencia CA, USA) incorporating the on-column DNase I digestion with the RNase-Free DNase Set (Qiagen) in the isolation procedure, according to manufacturer’s instructions. RNA concentration was determined spectrophotometrically using a NanoDrop 1000 (Thermo Scientific, Waltham, Massachusetts, USA) and agarose gel electrophoresis was used to ascertain RNA quality and integrity.

After checking RNA integrity and to rule out any possibility of DNA contamination before proceeding to RNA amplification, samples were subject to an additional DNase digestion treatment immediately before amplification using TURBO DNase (Ambion, Foster City CA, USA) according to manufacturer’s instructions. RNA amplification was performed using the Amino Allyl MessageAmp II aRNA Amplification Kit (Ambion). In brief, the reaction consisted of a reverse transcription step that generated a first-strand cDNA containing a T7 promoter sequence during a 2 h reaction at 42°C, followed by second-strand cDNA synthesis for 2 h at 16°C. After cDNA purification, *in vitro* transcription for 12–14 h at 37°C was used to generate antisense amplified RNA. A total of 100 ng total RNA was amplified in two sequential rounds of amplification where amino allyl UTP nucleotide incorporation was performed only during the second round of amplification, per the manufacturer’s instructions.

### Microarray experiment and signal acquisition

The PtGen2 cDNA microarray used in this study has been previously described [7] and additional information regarding the array can be obtained from NCBI’s Gene Expression Omnibus (GEO) under the Platform accession GPL11184. Experimental samples consisted of amplified RNA from zygotic embryos at each of the five time points shown in Figure 1A. Three biological replicates were analyzed for each time point and two technical replicates were conducted for each sample. The hybridization reference sample consisted of a pool containing equal amounts of total RNA from all five time points investigated. Fluorescent labelling, hybridization, pre- and post-hybridization washes, scanning, and data processing were performed according to Lorenz et al. [86], except that 2 μg of Cy-labeledamplified RNA was used to hybridize with the array after fragmentation using Fragmentation Reagent (Ambion, Cat. #AM8740). Microarrays were scanned using a ProScanArrayTM confocal scanner (Perkin Elmer, Waltham, MA) equipped with 532 nm and 635 nm lasers. Raw fluorescence data were processed using ImaGene (Bio-Discovery Inc., El Segundo, CA, USA).

Low-quality raw data and blank controls were filtered as previously described [7,87,88].

### Microarray data normalization and annotation

Filtered raw data was analyzed using the Bioconductor Limma package [89]. Data was normalized within array (Print-tip loess) and between arrays (Cyclic Loess) according to Smyth and Speed [90], and intensity ratios were transformed into log_2_ values. No background correction was applied and duplicated probes were treated independently during normalization.

Samples were clustered using the MeV program [91]. To incorporate updated transcript information for *P. pinaster*, the PtGen2 cDNA microarray (GPL11184) was re-annotated against sequences in the *P. pinaster* database, SustainPineDB (Version 2; http://www.scbi.uma.es/sustainpine), which contains 92 478 unique transcripts from maritime pine. The EST sequences representing both the 3′ and 5′ ends of the cDNA clones used to generate the array [5] were aligned using BLASTX (E-value 1e-10) to identify *P. pinaster* transcripts corresponding to each spot.

### Transcript profiles and functional category analysis

*P. pinaster* unigenes or ESTs were used as BLASTX queries to identify homologous sequences (E-value < 1e-10) in the NCBI protein (nr) or *A. thaliana* TAIR10 databases. Corresponding gene ontologies, enzymatic code numbers, plant ontology, pathway and gene family terms were collected using Blast2GO [92], which was also used for the enrichment analysis.

Differentially transcribed genes were identified from data for the different time points and replicates using the Bioconductor maSigPro package [22]. We defined a quadratic regression model to identify differentially transcribed genes across the five time points based on the premise that this model should have sufficient power to analyze a reduced number of time points [22]. A false discovery rate (FDR) of 0.01 was applied to identify significantly differentially transcribed genes. The maSigPro stepwise regression was adjusted to use the “two.ways.backward” method, with alfa 0.01 and an R^2^ cut-off value fit of 0.7. The use of high R^2^ gene models has been proposed for capturing biological meaningful expression trends and profile differences [22]. Significantly differentially transcribed genes were clustered using a hierarchical approach based on correlation distances. Unigenes not present in flowering plants (or other more distant *taxa*), but present in gymnosperms, were annotated as specific to gymnosperms. To make this identification, the complete list of proteins in the NCBI proteins (nr) database belonging to gymnosperm *taxa* (Tax IDs 58021, 3312, 58020 and 58022) was generated (GI list) and downloaded from NCBI Entrez database (104709 proteins). We used the BLASTX options *negative_gilist* or *gilist*, to respectively exclude or restrict comparisons to gymnosperms. Functional assessment was performed using maSigFun, which fits the regression model for groups of genes annotated to a similar functional category [21]. In this case, the groupings were based on gene ontology terms, enzyme code numbers, plant ontology terms, gene families and pathways, and a selection of other categories for which we had significant models.

Intensity values from the time-course microarray analysis of *A. thaliana* embryogenesis were available from the work of Xiang et al. (http://www2.bri.nrc.ca/plantembryo/) [16] and were normalized to obtain the expression values by subtracting a constant background value, as described by the authors. For the comparative analysis performed in our study, the *A. thaliana* globular, heart, torpedo, bent and mature embryo stages were considered equivalent to the Day0, Day5, Day11, Day15 and Day25 embryo samples from *P. pinaster*, respectively. For each gene, the fold-change between the transcript level values at each stage *versus* the average of the values in all the stages for each species was calculated. Since the *P. pinaster* unigene putative homologues in *A. thaliana* had been previously identified, transcript profiles were compared using Pearson correlation.

### Validation of microarray data using real-time RT-qPCR

RT-qPCR was used for independent verification of the transcript profiles of a sub-set of genes identified from the microarray data. Primers were designed with Primer3 program (http://primer3.sourceforge.net/) to have a Tm of 58°C, bind the gene conserved regions and amplify around 100 bp (Additional file 10). Reverse transcription was performed using cDNA synthesized from 1.25 μg of total RNA using the Transcriptor High Fidelity cDNA Synthesis Kit (Roche Diagnostics, USA) with the Random Hexamer primers according to the manufacturer’s instructions. Subsequent quantitative PCR (qPCR) was performed in a Roche LightCycler 480 system following standard cycling conditions. The SYBR Green I Master kit (Roche diagnostics, USA) was used to prepare the qPCR reaction mixtures, each one containing 1.8 μl of cDNA and 400 nM of each primer in a total volume of 20 μl. DNA contamination was discarded by the absence of signal after qPCR amplification of RNA samples with the *UBIQUITIN* primers. Negative controls (no template) were included in all the runs and the presence of only one specific peak was checked in the melting curve (dissociation curve). A pool containing equal amounts of total RNA from all five time points was included as a calibrator and positive control. Two biological replicates of each sample and three technical replicates per biological replicate were analyzed. The efficiency of each pair of primers was calculated based on the regression analysis of the PCR reactions kinetics by program LinReg 11.3 [93], and the Ct values by Roche Lightcycler software (Additional file 10). The relative amount of each transcript was calculated by the delta-delta-Ct method [94] using *UBIQUITIN* as reference gene, which has been probed reliable as endogenous control during *P. pinaster* somatic embryogenesis [95].

### Availability of supporting data

The data set supporting the results of this article is available in the NCBI GEO database, accession number GSE32551 (http://www.ncbi.nlm.nih.gov/geo/query/acc.cgi?acc=GSE32551).

## Competing interests

The authors declare that they have no competing interests.

## Authors’ contributions

MS was involved in the experimental design, sample collection and preparation, microarray hybridization, interpretation of the results and writing of the manuscript. JVB performed the bioinformatic analyses, participated in the interpretation of results and preparation of the manuscript. WWL supervised sample labelling, microarray hybridizations and took part in the critical review of the manuscript. ASR contributed for the validation of microarray data. RA contributed to the experimental design and provided inputs for interpretation of results. JFDD contributed materials and facilities and was involved in the critical review of the manuscript. CM conceived and supervised the project, participated in the interpretation of results, wrote and edited the manuscript. All authors have read and approved the final manuscript.

## Supplementary Material

Additional file 1 **Microarray data normalization and replicate clustering. **Box plot of the distribution of the red and green intensity ratio (M) from the thirty hybridized chip arrays before (A) and after (B) normalization. Click here for file

Additional file 2 **Microarray intensity values for the 30 slides after normalization and correspondence between spot, EST accession number and *Pinus pinaster *unigene.** Click here for file

Additional file 3 **Functional annotation of the *Pinus pinaster *unigenes. **Annotation was based on the homologous in NCBI and *Arabidopsis* databases, including gene putative prediction, gene ontology, pathways and families. Click here for file

Additional file 4 **Quality assessment of the gene annotation.** (A) Similitude values of the BLASTX alignments. (B) Species with the best BLASTX alignment of each query sequence. (C) Number of gene ontology terms per sequence for each group of terms. (D) Relation between number of gene ontology terms and length of the query sequence. Click here for file

Additional file 5 Functional annotation, specificity, clustering results and statistical analysis of the differentially expressed transcripts. Click here for file

Additional file 6 Clustering, fold-change and functional annotation of the differentially expressed transcripts in consecutive embryo stage-to-stage transitions. Click here for file

Additional file 7 **Correlation of gene expression during *A. thaliana *and *P. pinaster *embryogenesis.** The *A. thaliana* globular, heart, torpedo, bent and mature embryo stages [16] were considered equivalent to the Day0, Day5, Day11, Day15 and Day25 embryo samples of *P. pinaster*, respectively. For each sampling time, genes were plotted in a scatter graph using the *A. thaliana* (Y-axis) and *P. pinaster* (X-axis) expression values as coordinates. Click here for file

Additional file 8 **Correlation of the expression profiles between *A. thaliana *and *P. pinaster *along five embryogenesis stages.** Enrichment analysis values of the gene ontologies over/under-represented in the common subset of highly co-regulated transcripts. Click here for file

Additional file 9 **Enrichment analysis of the genes with a highly similar expression profile in *A. thaliana *and *P. pinaster.*** Click here for file

Additional file 10 Primers, efficiencies, Ct values and normalization of the target genes used for the data validation by qRT-PCR. Click here for file
