# Supplementary figures and images for: Transcriptomic analysis highlights epigenetic and transcriptional regulation during zygotic embryo development of Pinus pinaster
Source: BMC Plant Biol. 2013 Aug 30;13:123. doi: 10.1186/1471-2229-13-123 (PMC3844413; doi:10.1186/1471-2229-13-123)

**A**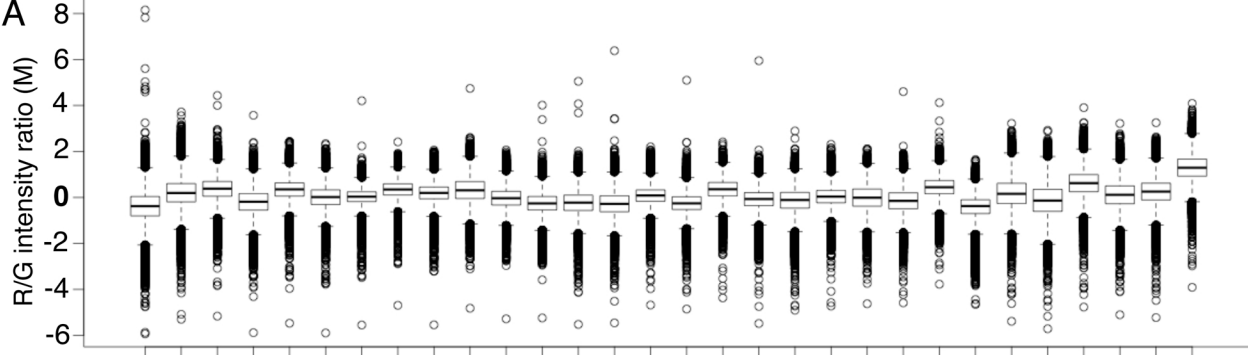**B**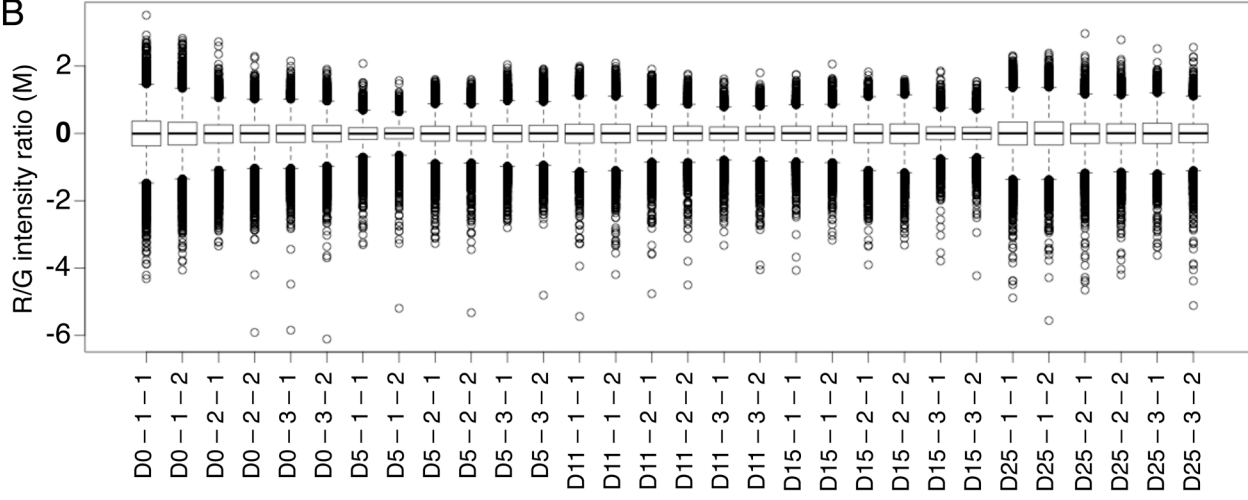

Supplement: Additional file 1 — Microarray data normalization and replicate clustering. Box plot of the distribution of the red and green intensity ratio (M) from the thirty hybridized chip arrays before (A) and after (B) normalization. [file 1471-2229-13-123-S1.pdf]

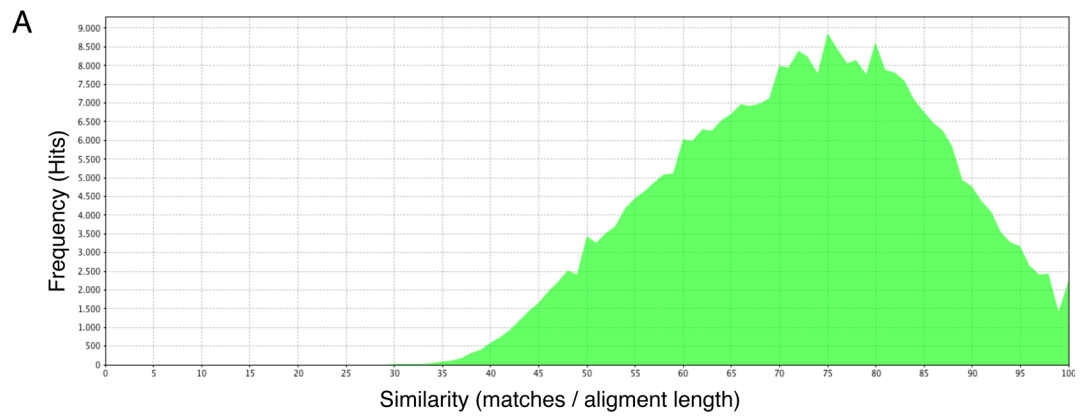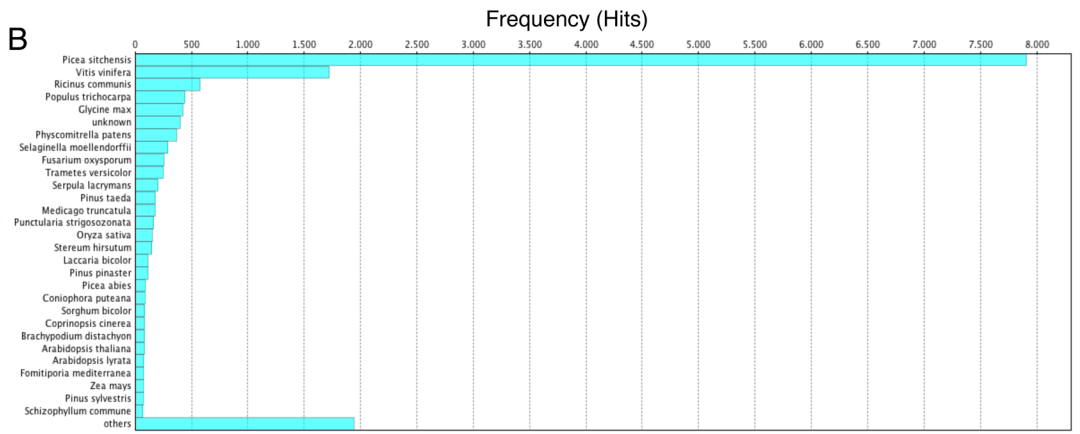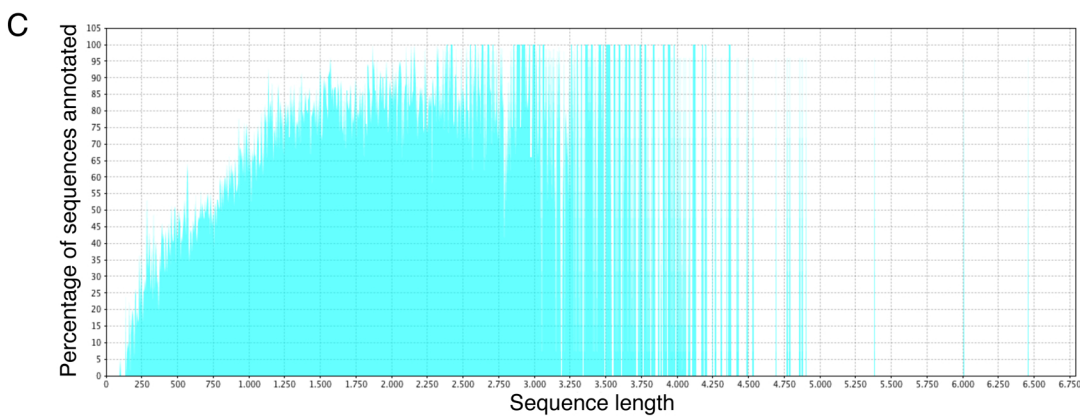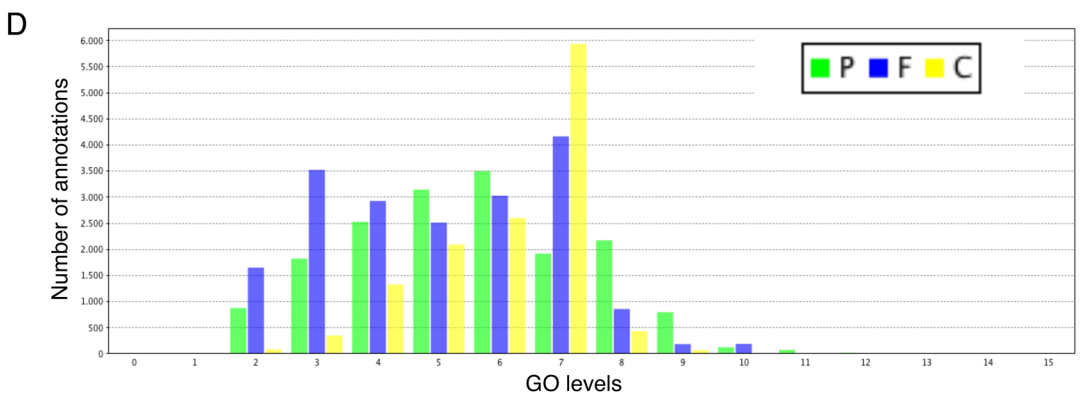

Supplement: Additional file 4 — Quality assessment of the gene annotation. (A) Similitude values of the BLASTX alignments. (B) Species with the best BLASTX alignment of each query sequence. (C) Number of gene ontology terms per sequence for each group of terms. (D) Relation between number of gene ontology terms and length of the query sequence. [file 1471-2229-13-123-S4.pdf]

*Arabidopsis thaliana*

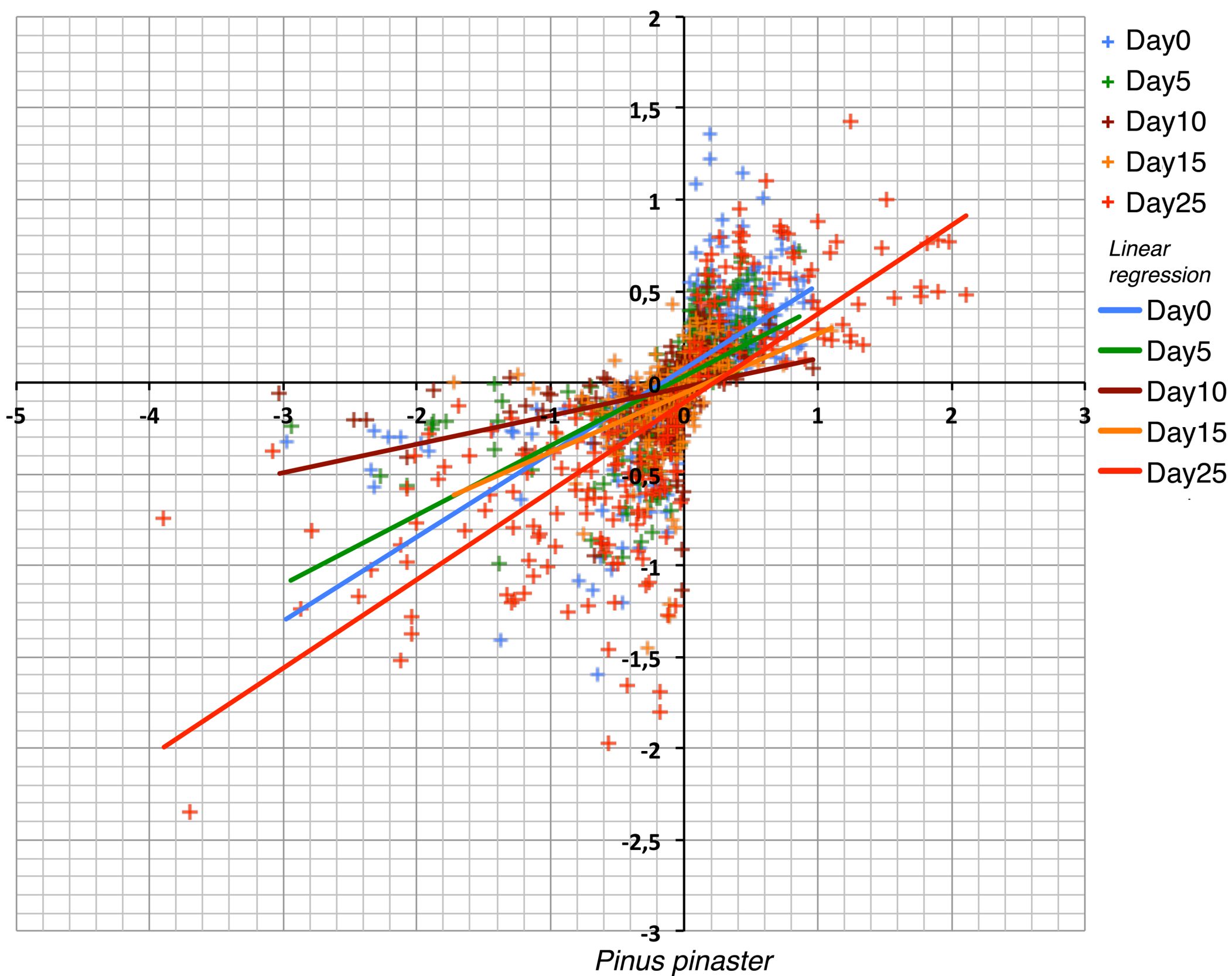

Supplement: Additional file 7 — Correlation of gene expression during A. thaliana and P. pinaster embryogenesis. The A. thaliana globular, heart, torpedo, bent and mature embryo stages [16] were considered equivalent to the Day0, Day5, Day11, Day15 and Day25 embryo samples of P. pinaster, respectively. For each sampling time, genes were plotted in a scatter graph using the A. thaliana (Y-axis) and P. pinaster (X-axis) expression values as coordinates. [file 1471-2229-13-123-S7.pdf]

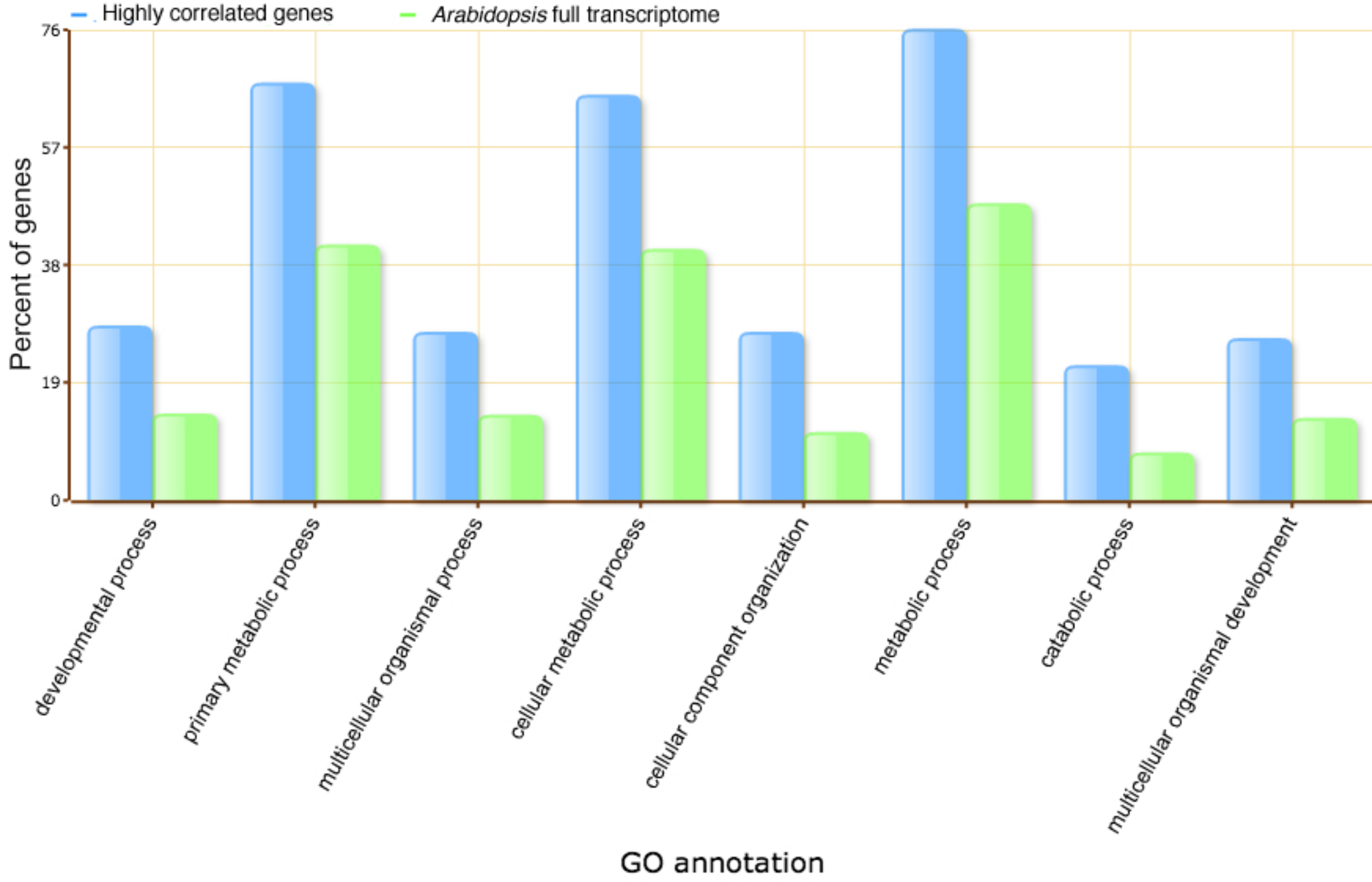

Supplement: Additional file 9 — Enrichment analysis of the genes with a highly similar expression profile in A. thaliana and P. pinaster. [file 1471-2229-13-123-S9.pdf]
